# Software for prioritizing conservation actions based on probabilistic information

**DOI:** 10.1111/cobi.13681

**Published:** 2021-07-16

**Authors:** Matthew Watts, Carissa J. Klein, Vivitskaia J. D. Tulloch, Silvia B. Carvalho, Hugh P. Possingham

**Affiliations:** ^1^ University of New England Armidale New South Wales Australia; ^2^ Australian Research Council Centre of Excellence for Environmental Decisions Brisbane Queensland Australia; ^3^ Centre for Biodiversity and Conservation Science University of Queensland St. Lucia Queensland Australia; ^4^ School of Earth Environmental Sciences University of Queensland St. Lucia Queensland Australia; ^5^ Conservation Decisions Lab, Department of Forest and Conservation Science University of British Columbia Vancouver British Columbia Canada; ^6^ Centro de Investigação em Biodiversidade e Recursos Genéticos da Universidade do Porto R. Padre Armando Quintas Vairão Portugal

**Keywords:** biodiversity, climate change, decision support, Marxan, optimization, probability, protected areas, simulated annealing, spatial conservation prioritization, species distribution modeling, apoyo a decidir, áreas protegidas, biodiversidad, cambio climático, Marxan, modelado de la distribución de especies, optimización, priorización de la conservación espacial, probabilidad, reconocimiento simulado, 生物多样性, 决策支持, Marxan 软件, 优化, 概率, 保护区, 模拟退火法, 空间优先保护, 物种分布建模, 气候变化

## Abstract

Marxan is the most common decision‐support tool used to inform the design of protected‐area systems. The original version of Marxan does not consider risk and uncertainty associated with threatening processes affecting protected areas, including uncertainty about the location and condition of species’ populations and habitats now and in the future. We described and examined the functionality of a modified version of Marxan, Marxan with Probability. This software explicitly considers 4 types of uncertainty: probability that a feature exists in a particular place (estimated based on species distribution models or spatially explicit population models); probability that features in a site will be lost in the future due to a threatening process, such as climate change, natural catastrophes, and uncontrolled human interventions; probability that a feature will exist in the future due to natural successional processes, such as a fire or flood; and probability the feature exists but has been degraded by threatening processes, such as overfishing or pollution, and thus cannot contribute to conservation goals. We summarized the results of 5 studies that illustrate how each type of uncertainty can be used to inform protected area design. If there were uncertainty in species or habitat distribution, users could maximize the chance that these features were represented by including uncertainty using Marxan with Probability. Similarly, if threatening processes were considered, users minimized the chance that species or habitats were lost or degraded by using Marxan with Probability. Marxan with Probability opens up substantial new avenues for systematic conservation planning research and application by agencies.

## Resumen

Marxan es la herramienta de apoyo a las decisiones que más comúnmente se usa para orientar el diseño de los sistemas de áreas protegidas. La versión original de Marxan no considera el riesgo y la incertidumbre asociados con los procesos amenazantes que afectan a las áreas protegidas, incluyendo la incertidumbre sobre la ubicación y la condición de las poblaciones de las especies y su hábitat ahora y en el futuro. Describimos y analizamos la funcionalidad de una versión modificada de Marxan: Marxan con Probabilidad. Este software considera explícitamente cuatro tipos de incertidumbre: probabilidad de que una característica exista en un lugar en particular (estimada con base en los modelos de distribución de especies o con modelos de población espacialmente explícitos); probabilidad de que las características de un sitio se pierdan en el futuro debido a un proceso amenazante, como el cambio climático, las catástrofes naturales y las intervenciones humanas descontroladas; probabilidad de que una característica existirá en el futuro debido a los procesos naturales de sucesión; como los incendios o las inundaciones; y probabilidad de que una característica exista pero haya sido degradada por los procesos amenazantes, como la sobrepesca y la contaminación, y por lo tanto no puede contribuir a los objetivos de conservación. Sintetizamos los resultados de cinco estudios que ilustraron cómo cada tipo de incertidumbre puede usarse para orientar el diseño del área protegida. Si hubiera incertidumbre en la distribución de la especie o de su hábitat, los usuarios podrían maximizar la posibilidad de que estas características estuvieran representadas mediante la inclusión de Marxan con Probabilidad. De manera similar, si los procesos amenazantes estuvieran considerados, los usuarios minimizarían la posibilidad de que se pierda la especie o degrade el hábitat usando Marxan con Probabilidad. Marxan con Probabilidad abre nuevas vías importantes para la investigación sobre la planeación sistemática de la conservación y su aplicación por parte de las agencias.

## 【摘要】

Marxan 软件是指导保护区系统设计的常用决策支持工具。然而, Marxan 的原始版本没有考虑到影响保护区的威胁过程的风险和不确定性, 包括物种种群和栖息地现在及未来位置和各种条件的不确定性。我们设计并检验了改进版 Marxan 软件的功能, 即考虑可能性的 Marxan (Marxan with Probability) 。该软件明确地考虑了四种不确定性:一种特征存在于特定地点的可能性 (基于物种分布模型或空间显式种群模型估计); 一个位点的特征由于未来气候变化、自然灾害, 或不受控制的人类干预等威胁过程而消失的可能性;一种特征由于自然的连续过程, 如火灾或洪水, 在未来存在的可能性;特征虽然存在, 但由于过度捕捞或污染等威胁过程而退化, 因此不能促进保护目标实现的可能性。我们总结了五项研究的结果, 来说明每种类型的不确定性如何用于指导保护区设计。如果物种或栖息地分布存在不确定性, 用户可以通过使用 Marxan with Probability 来纳入不确定性, 从而尽可能地表示出这些特征。类似地, 如果要考虑威胁过程, 用户也可以使用 Marxan with Probability 将物种或栖息地丧失或退化的可能性降至最低。 Marxan with Probability 这一软件为各机构的系统保护规划研究和应用开辟了重要新途径。**【翻译: 胡怡思; 审校: 聂永刚】**

## Introduction

Protected areas (e.g., national parks) are the cornerstone of most marine and terrestrial biodiversity conservation strategies worldwide (Possingham et al., 2006). In an attempt to achieve international, national, and local policy goals and associated biodiversity conservation targets (e.g., Convention on Biological Diversity (CBD) Aichi Target 11, [CBD, 2010]), Zero Draft of the Post‐2020 Global Biodiversity Framework [CBD, 2020]), protected areas are continuously being established or expanded to protect biodiversity (Tuvi et al., 2011), and the designation of new protected areas is likely to continue in the future. For instance, the Zero Draft of the Post‐2020 Global Biodiversity Framework explicitly states the goal of increasing coverage of protected areas to 30% of land and 10% of seas (CBD, 2020). The EU biodiversity strategy goes beyond these targets (at least 30% of both land and sea) (European Commission, 2011).

Over the past 25 years, systematic approaches and tools to support decisions about the location of protected areas have evolved rapidly (Daigle et al., 2020; Hanson et al., 2019; Margules & Pressey, 2000; Moilanen et al., 2009*a*). Optimization tools that use quantitative approaches to identify priorities are now commonly used because they can consider large amounts of spatial data (e.g., species and habitat distributions, socioeconomic costs) and they are repeatable and transparent (Moilanen et al., 2009*a*; Sinclair et al., 2018). There are 2 well‐accepted quantitative approaches and associated optimization tools used to identify spatial conservation priorities: minimum‐set problem and maximal‐coverage problem (Moilanen et al., 2009*a*). Marxan software is the most widely used software in protected area planning (Sinclair et al., 2018). It solves the minimum‐set problem, which is to achieve conservation targets (e.g., protect 20% of each habitat type) while minimizing the resources expended (Ball et al., 2009). To achieve this, it uses an algorithm to minimize an objective function with the following objectives: achieve the conservation targets, minimize the overall cost, and achieve a specified degree of spatial compactness. Marxan with Species Probability (or MarProb2D) and Marxan with Threat Probability (or MarProb1D) add additional constraints to this objective function: respectively, represent species with a specified minimum level of certainty and avoid threatened areas with low reliability.

Zonation software (Moilanen et al., 2009b) is also commonly used for protected area planning (Delavenne et al., 2012; Leathwick et al., 2008). It is used to solve the maximal coverage problem, as opposed to the minimum‐set problem that Marxan solves. The maximal coverage problem aims to maximize the biodiversity benefit for a fixed budget (Moilanen et al., 2009*a*). If used appropriately, Zonation and Marxan often deliver similar spatially explicit priorities for protected areas (Delavenne et al., 2012). Although these tools originally did not account for uncertainty, both have now been updated to explicitly include probabilistic information concerning occurrence, as well as other types of uncertainty (Klein et al., 2013; Moilanen et al., 2006; Tulloch et al., 2013).

A key component of all spatial prioritization problems is the adequate representation of biodiversity features in the protected area system (Possingham et al., 2006). These biodiversity features are often species or habitat types, although they can also be different hierarchical levels of the evolutionary continuum (Carvalho et al., 2017), biodiversity facets (Pollock et al., 2017), ecological and evolutionary processes (Hanson et al., 2020; Klein et al., 2009), and ecosystem services (Chan et al., 2006; Egoh et al., 2010). Often planners assume that conservation features are evenly distributed across their distribution (i.e., either present or absent), are in good condition, and will exist in the future. In some cases, these assumptions are made due to a lack of information. In many cases, however, more detailed data are available but, until recently, spatial conservation prioritization approaches that could explicitly consider these types of risk or uncertainty did not exist.

Originally, data on spatial uncertainty could not be used with Marxan. We examined the functionality of a modified version of Marxan: Marxan with Probability. It is one of several different new versions of the Marxan software. Marxan with Probability can be used to explicitly consider 4 types of spatial uncertainty: probability that a feature exists in a site (Marxan with Species Probability), estimated through species distribution models, spatially explicit population models, or habitat mapping accuracy (Akçakaya et al., 2004; Franklin & Miller, 2009; Peterson et al., 2011; Tulloch et al., 2013); probability that features at a site are destroyed in the future due to a threatening process (Marxan with Threat Probability), such as climate change (Parmesan, 2006; Powers et al., 2016) or future anthropogenic activities (Witt & Hammill, 2018); probability that the feature does not exist at a site in the future due to natural successional processes (Marxan with Species or Threat Probability), such as a fire or flooding (Drechsler et al., 2009); probability that the feature exists but is degraded by threatening processes, such as overfishing or pollution (Chung et al., 2019; Klein et al., 2013; Tulloch et al., 2016), or logging (Carrasco et al., 2020), and cannot contribute to conservation goals (Marxan with Species or Threat Probability).

The basic objective in Marxan with Probability is to ensure that each conservation feature is represented in the protected area system, above the conservation target, with a specified level of confidence. As part of the software development, we used the software in 5 protected‐area design problems (Carvalho et al., 2011; Game et al., 2008; Klein et al., 2013; Lourival et al., 2011; Tulloch et al., 2013) to test the ability of the software to incorporate the 4 different types of uncertainties described above (Table 1). In our selected case‐study articles, abbreviated versions of some aspects of Marxan with Probability are described. Here, for the first time, we mathematically describe all aspects of the Marxan with Probability software.

**Table 1:** Overview of 5 cases in which Marxan with Probability was used to design a protected‐area system.*

MarProb version	Probability considered	Example input	Case study
Marxan with Species Probability (MarProb 2D)	probability that a species exists, estimated with species distribution models or spatially explicit population models	species distribution models	Carvalho et al. (2011) designed reserves that maximize the chance species are represented under different climate warming scenarios. They used species distribution models to predict the probability of occurrence of different species at sites on the Iberian peninsula now and in the future. They found “no regrets” conservation areas, which are areas important for every time frame and uncertainly level.
Marxan with Species Probability (MarProb2D)	probability that a feature exists by considering the accuracy of habitat maps	habitat distribution models	Tulloch et al. (2013) designed reserves that account for uncertainty inherent in coral reef habitat maps. They used accuracy information describing the probability of occurrence of several habitat types derived from remote sensing modeled data validated by field surveys in Fiji. They found that consideration of habitat mapping accuracy leads to bigger reserve networks that have a high probability of achieving habitat conservation targets.
Marxan with Threat Probability (MarProb1D)	probability that features in a site are lost in the future due to a threatening process	catastrophic events	Game et al. (2008) identified marine reserves that have the least chance of missing conservation targets as a result of coral bleaching, an event that can be catastrophic for coral reef ecosystems. They estimated the average bleaching risk for each reef in the Great Barrier Reef between 2008 and 2100. They found that it is possible to improve the likely persistence of conservation features by 60% within reserve networks for just a 2% increase in cost.
Marxan with Threat Probability (MarProb1D)	probability that a feature exists in the future due to natural successional processes	successional processes	Lourival et al. (2011) designed reserves that have a high chance of representing 20% of 5 different vegetation succession stages in 50 years. They modeled the probability of observing 5 different seral stages across the Patanal wetlands in South America given hydrological disturbance and successional ecosystem dynamics. They found that high priority areas differed when reserves were designed considering flood disturbances and succession dynamics compared to reserves designed based on static vegetation distributions.
Marxan with Threat Probability (MarProb1D)	probability the feature exists but is degraded by threatening processes and cannot contribute toward conservation goals	habitat degradation	Klein et al. (2013) identified marine reserves that have a high probability of being in good condition in California. They estimated the probability a site is in good condition based on human impacts that cannot be managed through reservation of the ocean, such as nutrient and sediment run‐off and invasive species. They found that increasing the probability of protecting good condition habitats in reserves from 50% to 99% costs fishers an additional 1.7% of their income.

* Collectively, the cases include the 4 different types of spatial uncertainty that the software can accomidate.Types of spatial uncertainty: probability a feature exists (estimated with species or habitat distribution models and spatially explicit population models); probability features in a site are lost in the future due to a threatening process (e.g., catastrophic events); probability a feature exists in the future due to natural successional processes (e.g., succession); and probability feature exists but is degraded by threatening processes (e.g., habitat degradation).

## Methods

The risks and uncertainties that Marxan with Probability can deal with could be associated with the sites where the features occur, for example, where all conservation features are lost from a site, or the individual features in each site, for example, where there are probabilistic species distribution models, over a particular time frame. We begin with defining the problem Marxan solves, which is essential to our explanation of the problem Marxan with Probability solves. All versions of Marxan use the simulated annealing algorithm to find several good solutions to the protected‐area design objective (Ball et al., 2009).

### Definition of the Problem Marxan Solves

Marxan delivers decision support for the design of spatial conservation areas, such as protected areas systems. The objective of Marxan is to identify a specified amount of each conservation feature (e.g., species and habitats) for a minimum cost. A geographic region of interest is subdivided into a number of planning units, which can be of any size or shape, and each planning unit is assigned a cost to reflect the cost of its protection. Each planning unit can be included in the set of selected planning units or excluded from the set. If a planning unit is included in the set, the conservation features the planning unit contains and the cost of the planning unit are added to the total feature representation and total cost of the set of selected planning units.

The mathematical problem to which Marxan finds solutions is (Ball et al., 2009; Watts et al., 2009),

(1) $$minimize\sum_{i}^{N}c_{i}x_{i}+b\sum_{i}^{N}\sum_{h}^{N}x_{i}\left(1-x_{h}\right)v_{ih}$$

subject to the constraint that all representation targets are met,

(2) $$\sum_{i=1}^{N}r_{ij}x_{i}\geq T_{j}\;\;\;\forall \;j,$$

where *x_i_* is a control variable indicating whether the planning unit (*i =* 1*… N*) is selected for reservation (*x_i_* = 1) or not (*x_i_* = 0), *r_ij_* is the occurrence level of feature *j* (*j =* 1*…M)* in site *i*, and *T_j_* is the target level for feature *j*. The first term of Eq. 1 is a penalty associated with the costs, *c_i_*, of all the selected planning units. In a typical planning problem, the cost might represent the financial cost of acquiring and managing a planning unit or it might represent the opportunity cost of activities displaced by selecting a planning unit (Ban & Klein, 2009; Naidoo et al., 2006). The second term of Eq. 1 is the penalty associated with the configuration of selected planning units, where v*_ih_* is the length of the boundary (or degree of positive connection) between planning units *i* and *h*, and *b* is a boundary multiplier which determines the cost of the protected area system relative to the penalty for its spatial configuration. This term thus controls for the aggregation level of the planning units selected. The index *h* iterates over the same planning units as the index *i*.

To solve the problem, an objective function is created by transforming the constraints in Eq. 2 into an additional penalty term that is added to Eq. 1. The additional penalty term is

(3) $$y\sum_{j=1}^{M}F_{j}R_{j}\left(1-H_{s}\left(\frac{\sum_{i=1}^{N}r_{ij}x_{i}}{T_{j}}\right)\right),$$

where *y* is a weighting value that controls the relative importance of the different terms in the objective function, in this case the cost of missing targets; *F_j_* is the feature penalty factor that indicates the relative importance of meeting the representation target, *T_j_*, for feature *j*, *R_j_* is the additional cost of meeting the representation target of feature *j*. The *F_j_* value is provided by the user and can be determined through an iterative calibration process in which the user balances the importance of meeting the representation target of the feature against the importance of minimizing the overall cost of the selected planning units. Game and Grantham (2008) provide an extensive discussion of the procedure for calibrating *F_j_*. The term *R_j_* is computed by Marxan as the representation cost of meeting the representation target of feature *j* (Watts et al., 2009). The Heaviside function, *H(* **x**), is a step function that takes a value of 0 when **x** *≤* 1 and 1 otherwise. In essence, once the target is met for a species, the penalty for that species becomes 0.

### Definition of the Problem Marxan with Probability Solves

**Figure 1:**
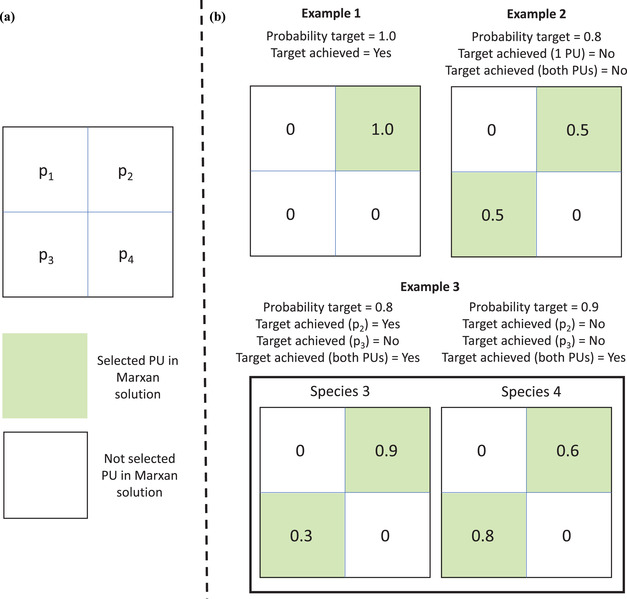
Simplified example of how Marxan with Probability selects planning units: (a) a hypothetical study area with 4 planning units (PU) (p1, p2, p3, and p4) (green, selected units; white, units not selected) and (b) 3 examples of data sets containing probabilities of occurrence of different species in each of 4 planning units (examples 1 and 2, 1 species occurs in the study area; example 3, 2 species occur in the study area). Calculations done in Marxan with Probability to determine the probability of representing at least 1 occurrence of each species, with a specific probability target, under the selected planning units are described in the text. For all 3 examples, the target for each species was 1

The objective of Marxan with Probability is to ensure features meet their conservation targets with a specified level of security in a protected area system where entire sites and all the features in them, or individual features in a site, can be lost even when represented in the protected area system. Marxan with Probability solves a problem similar to the problem that Marxan solves. The difference is the deterministic constraint is replaced on meeting a target (Eq. 3), with a probabilistic constraint:

(4) $$p_{j}\left(x,T_{j}\right)\geq P_{j}\;\forall j,\;where\;0<p_{ij}<1,$$

where $p_{j}\left(x,T_{j}\right)$ is the probability that feature *j* meets the target (*T_j_*) given protected area system **x**, and *P_j_* is the level of certainty with which one wishes to meet that target (ptarget1D for Marxan with Threat Probability and ptarget2D for Marxan with Species Probability [Appendix S1]). As with the representation target constraint, the probability constraint is implemented through a new penalty term in the objective, henceforth referred to as the probability penalty. Similar to the other penalty terms (e.g., penalty for not representing biodiversity features), the probability penalty term can be weighted to control its importance relatively to other penalties. To calculate the probability penalty, Marxan predicts the probability that each feature contained in a protected area system will be exposed to threatening processes (Marxan with Threat Probability) or represented within the protected area system with a given level of certainty (Marxan with Species Probability) and then sums the probabilities of all features:

(5) $$w\sum_{j=1}^{N_{f}}F_{j}R_{j}H\left(P_{j}-p_{j}\left(x,T_{j}\right)\right)\left(\frac{P_{j}-p_{j}\left(x,T_{j}\right)}{P_{j}}\right),$$

where *w* is the weighting value that controls the relative importance of the probability penalty in the objective function (ptarget1D or ptarget2D [Appendix S1]). For each conservation feature, a penalty, *R_j_*, is imposed when the probability target, *P_j_*, is not achieved, and it is proportional to the shortfall in achievement of the probability target for feature *j* (Appendix S1). The Heaviside function, *H(* $P_{j}-p_{j}\left(x,T_{j}\right)P_{j}$ *)*, takes a value of 0 when the probability of capturing the species target is above the desired level of reliability (i.e., $P_{j}-p_{j}\left(x,T_{j}\right)$<0) and 1 otherwise (Appendix S1). Simple examples are provided in Figure 1 that illustrate how Marxan with Probability works.

In Figure 1, example 1, the probability that species 1 occurs in *p*_2_ is 1. The probability the feature occurs in *p*_1_, *p*_3_, and *p*_4_ is 0. The probability target for species 1 is 1.0. The probability that the reserve network contains at least one occurrence of the species, *p*_yes_. The probability the reserve network does not contain a single occurrence of the feature can be calculated with *p*_no:_ *p*_no_ = 1 − *p*_2_ = 0 and *p*_yes_ = 1 − *p*_no_ = 1 − (1 − *p*_2_) = 1. If Marxan selects a single planning unit, it will select *p*_2_. Because *p*_yes_ is 1, one can be certain the reserve network will contain at least 1 occurrence of the feature.

In Figure 1, example 2, the probability that species 2 occurs in *p*_2_ and *p*_3_ is 0.5. The probability the feature occurs in *p*_1_ and *p*_4_ is 0. The probability target for species 1 is 0.8. If 1 planning unit is selected, either *p*_2_ or *p*_3_ will be selected, and *p*_no_ and *p*_yes_ is calculated as follows: *p*_no_ = 1 − *p*_2_ = 0.5 or equivalently *p*_no_ = 1 − *p*_3_ = 0.5 and *p*_yes_ = 1 − *p*_no_ = 0.5.

Thus, for either planning unit, the probability of the species being present in the protected areas system is below the probability target (0.8). If 2 planning units are selected, *p*_2_ and *p*_3_ will be selected, and *p*_no_ and *p*_yes_ are calculated as follows: *p*_no_ = (1 − *p*_2_) (1 − *p*_3_) = 0.5 ^*^ 0.5 = 0.25 and *p*_yes_ = 1 − *p*_no_ = 0.75. In this case, because the probability target of the species is above 0.75, the probability target for this species will not be achieved and a penalty is added to the objective function. This differs from the standard version of Marxan, which is based on the assumption the species exists in both planning units and does not add a penalty to the objective function.

In Figure 1, example 3, the probability that species 3 occurs in *p*_2_ is 0.9 and in *p*_3_ is 0.3. The probability the feature occurs in *p*_1_ and *p*_4_ is 0. The probability target for species 3 is 0.8. The probability that species 4 occurs in *p*_2_ is 0.6, in *p*_3_ is 0.8, and in *p*_4_ is 0.1. The probability the feature occurs in *p*_1_ is 0. The probability target for species 4 is 0.9. If 1 planning unit is selected, either *p*_2_ or *p*_3_ will be selected, and *p*_no_ and *p*_yes_ is calculated as follows:

If *p*_2_ is selected, *p*_no (species3)_ = 1 − *p*_2(species3)_ = 0.1, *p*_yes (species3)_ = 1 − *p*_no(species3)_ = 0.9, *p*_no (species4)_ = 1 − *p*_2(species4)_ = 0.4, and *p*_yes (species4)_ = 1 − *p*_no (species4)_ = 0.6. In this case, the probability target would be achieved for species 3 (>0.8), but not for species 4 (>0.9). Thus, a probability penalty would be added to the objective function: shortfall species 3 = 0, shortfall species 4 = 0.3, and probability penalty = shortfall species 3 + shortfall species 4 = 0.3. The probability weight determined by the user would then be multiplied by the probability penalty and balanced against the other terms of the objective function. Similarly, if *p*_3_ is selected, *p*_no (species3)_ = 1 − *p*_3(species3)_ = 0.7, *p*_yes (species3)_ = 1 − *p*_no(species3)_ = 0.3, *p*_no (species4)_ = 1 − *p*_2(species4)_ = 0.2, and *p*_yes (species4)_ = 1 − *p*_no (species4)_ = 0.8. In this case, the probability target would not be achieved for species 3 (<0.8), nor for species 4 (<0.9). If the value of the probability term is too high, Marxan adds another planning unit to the protected areas system.

In the case that both *p*_2_ and *p*_3_ are in the protected areas system, the probability of representing each of the species would be the following: *p*_no (species3)_ = (1 − *p*_2(species3)_) (1 − *p*_3(species3)_) = 0.07, *p*_yes (species3)_ = 1 − *p*_no(species3)_ = 0.93, *p*_no (species4)_ = (1 − *p*_2(species4)_) (1 − *p*_3(species4)_) = 0.08, and *p*_yes (species4)_ = 1 − *p*_no (species4)_ = 0.92. In this case, the probability target would be achieved for both species, thus no penalty would be added to the objective function.

Calculating the exact probability that feature *j* meets target *T_j_*, given protected area system **x**, $p_{j}\left(x,T_{j}\right)$, can be computationally extremely expensive when the study region contains a large number of planning units, and is thus incompatible with the simulated annealing algorithm that Marxan uses to find good protected area system designs. Consequently, we developed a fast method of approximating the probability that any feature meets its conservation target with a predefined level of certainty for a particular protected area system.

### Statistical Approximation for $p_{j}\left(x,T_{j}\right)$

Calculating the precise probability of having less than the target amount of each feature remaining in the protected area system at the end of the planning horizon must be calculated quickly to be of use in protected area design. To do this, we approximated the chance each conservation feature would fail to meet its target with the central limit theorem and the standard normal distribution (Rice, 1995). We assumed the probability of having successfully conserved different amounts of each feature in the entire protected area system is normally distributed because it is the sum of many random variables. The mean is based on the expected amount of each feature conserved for a particular protected area system (Appendix S1), given by

(6) $$\mu_{j}=\sum_{i=1}^{N}r_{ij}x_{i}\left(1-q_{ij}\right)\;\forall \;j$$

and the variance in the expected amount of a feature contained in a protected area system is

(7) $$\sigma_{j}=\sqrt{\sum_{i=1}^{N}r_{ij}r_{ij}x_{i}q_{ij}\left(1-q_{ij}\right)}\;\forall \;j,$$

where *q_ij_* is the probability that the feature *j* is not actually in planning unit *i* in the time frame of interest due to one of the many processes described above (Appendix S1).

The standard score (i.e., *Z* score) of a feature contained in a protected area system (Appendix S1) indicates by how many standard deviations an observation or datum is above or below the mean and is calculated as:

(8) $$z_{j}=\frac{T_{j}-\mu_{j}}{\sigma_{j}}\;\forall \;j.$$

The software then determines the percentile rank from the standard score and statistical tables by applying a function, PrZUT (raw1D/2D) (Appendix S1), to derive the probability of feature capture in the protected area network (Marxan with Species Distribution) or probability that features are not exposed to threatening processes in the protected area network (Marxan with Threat Distribution) as follows:

(9) $$p_{j}\left(x,T_{j}\right)=PrZUT\left(z_{j}\right)\;\forall \;j.$$

Hence, the objective function of Marxan with Probability is to minimize

(10) $$\begin{array}{ccc} & & \sum_{i}^{N}c_{i}x_{i}+b\sum_{i}^{N}\sum_{h}^{N}x_{i}\left(1-x_{h}\right)v_{ih}+\;w\sum_{j=1}^{M}F_{j}R_{j}\\ & & \;\times \left(H\left(P_{j}-p_{j}\left(x,T_{j}\right)\right)\left(\frac{P_{j}-p_{j}\left(x,T_{j}\right)}{P_{j}}\right)\right). \end{array}$$

We considered 5 cases in which Marxan with Probability was used to design marine or terrestrial protected area systems to demonstrate the different types of spatial uncertainty that can be considered in Marxan with Probability. We chose these case studies because they were used to develop and system test Marxan with Probability and they feature a range of different conservation problems. The articles in which these cases are delineate describe the Marxan with Probability objective function in abbreviated form and in some instances have detailed mathematical information in online appendices (see Lourival et al., [2011] and Tulloch et al., [2013]).

The conservation planning software we wrote is freely available. It considers uncertainty in data and risks of events and describes the mathematical formulation of Marxan with Probability. The software is 4MB and is available from https://marxansolutions.org/ , which requires either Linux, MacOSX, or Microsoft Windows to use. The source code is available from https://github.com/mattwatts/marxan.

## Summary of Case Studies

To maximize the chance that species were represented given uncertainty in their distribution, Carvalho et al. (2011) used Marxan with Probability to include the probability of species’ occurrence under climate change in their reserve design, estimated using species distribution models (Table 1). Similarly, to maximize the chance that habitats were represented in a protected area system given uncertainty in their distribution, Tulloch et al. (2013) used Marxan with Species Probability to design a reserve system that included coral reef habitat mapping accuracy or error associated with remotely sensed mapped habitat data as the probability of occurrence (Table 1). By including uncertainty in species or habitat distributions, both approaches account for a certain level of risk in underrepresenting features in conservation planning.

To account for the probability that features in a site may be lost in the future due to a threatening process, Game et al. (2008) used Marxan with Probability to include the probability that reefs will be destroyed through catastrophic coral bleaching in their reserve design (Table 1). Thus, the approach can be used to plan for species persistence given future threats, as well as to identify trade‐offs between risk and costs of conservation.

To account for the probability that a feature exists in the future due to natural successional processes, Lourival et al. (2011) used Marxan with Probability to incorporate landscape spatiotemporal dynamics for 5 plant communities in a reserve design (Table 1). This approach to accounting for uncertainty can be used to optimize the current and future representation of species or communities given disturbance risk with maximum reliability (i.e., smallest uncertainty).

Finally, Klein et al. (2013) used Marxan with Probability to account for the probability the feature exists but is degraded by threatening processes by including information on whether a coastal marine site is in good condition based on the impacts of human activities using Table 1. This approach can be used to minimize the chance that final reserves include features in poor condition and avoid areas that are important commercially (e.g., for fishing).

## Discussion

We explicitly defined the new functionality of the Marxan software, which will open up new avenues of research and application for target‐based conservation planning. We feature a few of the many possible applications of Marxan with Probability, each of which demonstrates one of the 4 different types of spatial uncertainty that can be considered in the software (Table 1). Although our case studies focus on protected area design, the software could also be used to support decisions about the location of other conservation actions, not just protected areas, relevant to other catastrophic threats (e.g., salinity inundation), successional events (e.g., fire), and types of uncertainty (Plumptre et al., 2014).

Marxan with Probability allows conservation planners to use more detailed data and conduct more sophisticated analyses than more traditional spatial planning. For example, with few exceptions (Carvalho et al., 2010; Moilanen et al., 2006; Tulloch et al., 2013), probabilistic data from species and habitat distribution models are commonly converted to presence‐absence data based on a threshold (Fleishman et al., 2003), resulting in lost information. Notably, conserving 6 planning units where the probability of occurrence of a species is 50% in each planning unit is very different from protecting 3 sites where one is sure the species is present. Researchers who have used probabilistic distribution data in protected area design could not ensure that each conservation feature would be represented in the protected area system with a specified level of confidence, which is fundamental to Marxan with Probability. Further, Marxan with Probability offers a new way to consider threatening processes, which gives planners the ability to assess trade‐offs between threatening processes and conservation costs when designing protected areas (Game et al., 2008; Klein et al., 2013). Prior to the development of this software, if planners wanted to avoid threatening processes when designing protected areas, they would often ignore conservation costs and use a site's threat status as a substitute for a site's cost (Ban & Klein, 2009). Given the importance of considering both costs and threats in conservation planning, this approach is not appropriate when applied to real‐world conservation problems.

Climate change is one of the biggest threats to biodiversity (Pecl et al., 2017; Urban, 2015). Planning for conservation in a changing climate is a large area of research, and many sophisticated planning approaches have been developed (Jones et al., 2016; McLeod et al., 2009). Marxan with Probability offers new approaches to planning under climate change. It is especially useful for dealing with the uncertainty around the exact response of species and habitats to climate change, which may vary from place to place. It can accommodate data from future species distribution models (Carvalho et al., 2011) as well as models that estimate the likelihood of a threatening process associated with climate change (e.g., coral bleaching [Game et al., 2008]).

Marxan with Probability improves on standard prioritization approaches by accounting for uncertainty. A limitation of the approach is that Marxan with Probability assumes statistical independence of planning units and does not adjust for spatial autocorrelation. This will inflate the estimated probabilities, but the importance of this limitation will vary depending on how much weight users apply to the XXX; more clustering results in higher inflation. Although it is possible to include covariation in events in the mathematical formulation of Marxan with Probability, relevant data for such an application are rarely available. Further, the complexity of the approach would increase exponentially, making it difficult for managers to apply to inform conservation decisions. Furthermore, previous research demonstrates that Marxan with Probability solutions generally avoid planning units with high risk or uncertainty (Carvalho et al., 2011; Game et al., 2008*b*) because they can be replaced with sites that have higher chances of conserving those same features. If the objective is to protect biodiversity that is at higher risk, users would need to lower the certainty target, resulting in trade‐offs with solutions that are larger overall (Game et al., 2008*a*; Tulloch et al., 2013).

There have been other advances in Marxan, including the ability to plan for multiple conservation and resource use zones (Watts et al., 2009) and asymmetric connectivity (Beger et al., 2010; Daigle et al., 2020). The next step is to develop a version of Marxan that can integrate all the advances of Marxan so that one can simultaneously consider probabilistic data, multiple zones, and asymmetric connectivity. Although this would be useful for solving some types of conservation problems, it will require a great deal of data and be analytically complex, making it inaccessible or inappropriate for many common types of conservation problems. Regardless, any version of Marxan should be used to support, not make, conservation decisions and should only be used in conjunction with other stakeholder engagement and decision‐making processes.

## Supporting information

Appendix Table S1. The missing values file for MarProb1D (Marxan with Threat Probability)/MarProb2D (Marxan with Species Probability) has 8 additional fields compared to the standard Marxan missing values file.
