# Supplementary material for: Software for prioritizing conservation actions based on probabilistic information
Source: Conserv Biol. 2021 Jul 16;35(4):1299–308. doi: 10.1111/cobi.13681 (PMC8419856; doi:10.1111/cobi.13681)
Supplement: Supplementary file 1 — Appendix Table S1. The missing values file for MarProb1D (Marxan with Threat Probability)/MarProb2D (Marxan with Species Probability) has 8 additional fields compared to the standard Marxan missing values file. [file COBI-35-1299-s001.docx]

**Appendix 1**

**New Inputs**

Input.dat – requires new “probability weighting” field


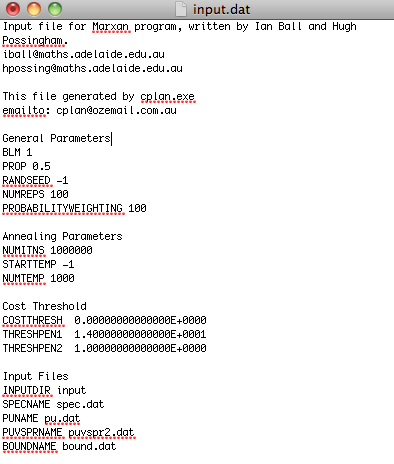


pu.dat – requires probability field (for Marxan with Threat Probability, or Marprob 1D)


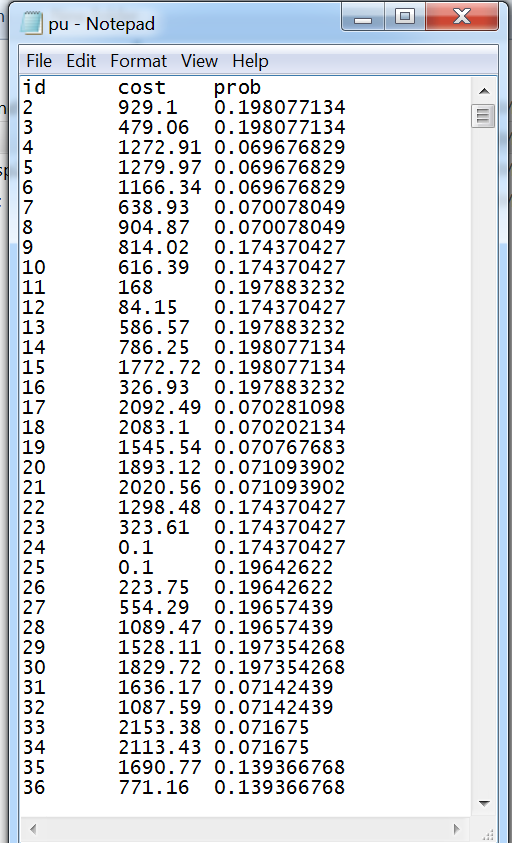


puvsp.dat – requires probability field (for Marxan with Species Probability, or Marprob 2D)


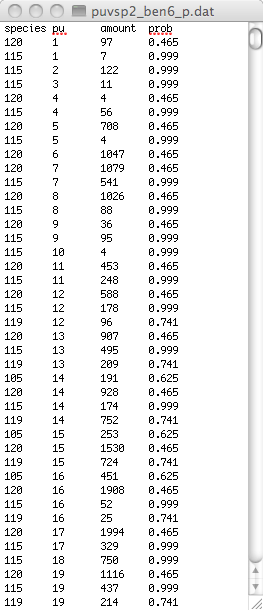


Spec.dat – requires ptarget1d (Threat probability) or ptarget2d (Species probability) field (the example given below shows ptarget2d, this would be changed to ptarget1d if using the threat probability version of Marxan)


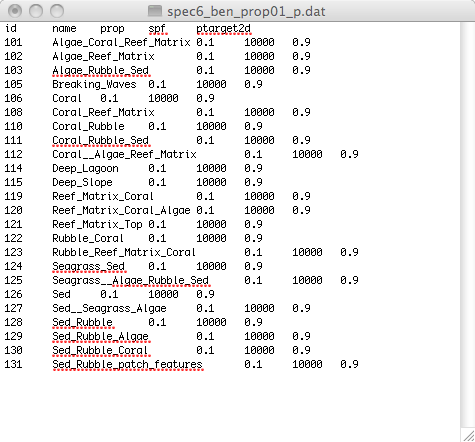


Appendix Table S1. The missing values file for MarProb1D (Marxan with Threat Probability)/MarProb2D (Marxan with Species Probability) has 8 additional fields compared to the standard Marxan missing values file.

| Field Name | Description |
| --- | --- |
| ptarget1d  or  ptarget2d | Threat avoidance target for each feature, as specified by the user in spec.dat  Areal representation target for each feature, as specified by the user in spec.dat |
| EA1D / EA2D | Expected Amount of a feature contained in a protected area network |
| VIEA1D / VIEA2D | Variance in Expected Amount |
| Z1D / Z2D | The Standard Score (or Z-score) |
| rawP1D / rawP2D | Probability of feature capture or threat avoidance as returned by probZUT |
| heavisideSF1D / heavisideSF2D | Binary value for the Heaviside function - a step function which takes a value of zero when the shortfall <=0 and 1 otherwise. The Heaviside step function ensures the penalty for each feature becomes zero when the predicted probability of threat avoidance is greater than the threat avoidance target (for MarProb 1D, or ensures the penalty for each feature becomes zero when the predicted probability of capture is greater than the capture target (for MarProb 2D) |
| shortfallP1D  shortfallP2D | Shortfall in the probability of threat avoidance (ptarget1d - rawP1D). It equals 1 when the threat avoidance target for each feature is not met within the configuration and approaches 0 as the predicted probability of threat avoidance approaches the threat avoidance target.  Shortfall in the probability of feature capture for each feature (ptarget2d – rawP2D. It equals 1 when the feature capture target for each feature is not met within the configuration and approaches 0 as the predicted probability of feature capture approaches the feature capture target |
| P1D / P2D | Probability term of the objective function for this feature (heavisideSF1D * shortfallP1D) |
